# Protective effects of biogenic selenium nanoparticles synthesized by *Chlorella Vulgaris* against bisphenol a-induced metabolic disturbances in rats

**DOI:** 10.1186/s11671-026-04715-2

**Published:** 2026-07-13

**Authors:** Mohamed A. Kandeil, Eman T. Mohammed, Rawya G. ELabd, Abdel‑Razik H. Abdel‑Razik, Dina Sabry, Doaa Sh. Mohamed

**Affiliations:** 1 Department of Biochemistry, Faculty of Veterinary Medicine, Beni-Suef University, Beni Suef, 62511 Egypt; 2 Department of Histopathology, Faculty of Veterinary Medicine, Beni-Suef University, Beni Suef, 62511 Egypt; 3 Department of Medical Biochemistry and Molecular Biology, Faculty of Medicine, Badr University in Cairo, Cairo, 11829 Egypt; 4 Department of Medical Biochemistry and Molecular Biology, Faculty of Medicine, Cairo University, Cairo, 11562 Egypt

**Keywords:** BPA, Green nanotechnology, *Chlorella vulgaris*, Biogenic selenium nanoparticles, metabolic disturbances

## Abstract

**Background:**

Bisphenol A (BPA), a xenoestrogen and endocrine disruptor, is widely used in plastic and polycarbonate products, contributing to metabolic disorders such as dyslipidemia and obesity. Algae, like *Chlorella vulgaris* (CV), contain highly active compounds that act as natural reducing agents, converting inorganic selenium into nanoparticle form.

**Methods:**

In this study, we developed novel biologically synthesized selenium nanoparticles using CV (SeNPs-CV) and investigated their protective properties in comparison to physically synthesized selenium nanoparticles (SeNPs) against BPA-induced metabolic disturbances in rats. 40 male albino rats were allocated into four groups (*n* = 10) and received different oral treatments for 45 days: a control group, a BPA group (150 mg/kg/day), a SeNPs group (2 mg/kg/day alongside BPA), and a SeNPs-CV group (2 mg/kg/day alongside BPA).

**Results:**

BPA exposure caused oxidative stress and various metabolic disturbances, evidenced by changes in redox biomarkers, upregulation of fatty acid synthesis-related genes (SREBP1, FAS, ACC1, miRNA-122), and altered lipid profiles. Levels of leptin, adiponectin, NF-κB, TNF-α, and liver function markers were also affected. However, co-treatment with SeNPs and SeNPs-CV significantly alleviated these alterations caused by BPA. Histopathological analysis corroborated these biochemical findings, showing improved hepatic tissue structures with SeNPs and SeNPs-CV.

**Conclusions:**

Overall, SeNPs-CV exhibit hypolipidemic, anti-inflammatory, and antioxidant properties that are on par with those of their physically synthesized equivalent, demonstrating their therapeutic potential and acting as an eco-friendly agent that promotes green innovation and environmental sustainability.

## Introduction

Bisphenol A (BPA) is frequently used in plastic products such as food containers, medical devices, baby bottles, and the lining of food cans [1]. BPA is among the first identified obesogenic endocrine disruptors. Growing evidence links BPA exposure to the development of metabolic disorders, involving atherogenic dyslipidemia, hypertension, atherosclerosis, cardiovascular diseases, insulin resistance, and obesity [2]. BPA also induces oxidative stress, which plays a major role in metabolic disorders by disrupting lipid metabolism and increasing the risk of cardiovascular diseases [3]. This oxidative stress results in lipid peroxidation, elevates reactive oxygen species (ROS), and activates various inflammatory pathways [4, 5]. Furthermore, obesity contributes to oxidative stress through chronic inflammation, while adipokines produced by fat cells increase ROS production, interfere with carbohydrate and lipid metabolism, and stimulate insulin resistance [6]. Given its widespread use and potential health risks, BPA remains a critical focus of scientific and regulatory concern.

Selenium (Se) is an essential trace element critical for redox homeostasis, immune response, and neurotransmission [7]. It plays a key role in selenoproteins like glutathione peroxidase (GPx) and selenocysteine, which are integral to antioxidant systems [8]. The use of selenium nanoparticles (SeNPs) in biomedical applications has expanded significantly, largely due to their high bioavailability, favourable biocompatible nature, and lower toxicity. They exhibit antimicrobial, antioxidant, and anti-inflammatory activities, making them useful for treating metabolic disorders like hyperlipidemia and hyperglycemia in diabetic rats [9]. SeNPs could be manufactured by chemical, physical or biological methods (also known as the “green synthesis”), with biologically synthesized SeNPs showing better compatibility with human tissues [10].

Green synthesis using plants and microorganisms, including algae, fungi and bacteria as biological catalysts, offers an eco-friendly method for SeNP production, eliminating the need for chemical agents [11]. Algal extracts, rich in bioactive compounds like flavonoids, terpenes, tannins, coumarins, and phenolic acids, serve as reducing and stabilizing agents during the synthesis of nanoparticles. These bioactive compounds exhibit antioxidant, anti-inflammatory, and insulin-sensitizing properties, further enhancing the biomedical applications of SeNPs [9]. The antioxidant properties of biogenic SeNPs are greater than those of sodium selenite, making biogenic synthesis a harmless and scalable production approach [12].

Amongst several biological methods proposed for the green synthesis of SeNPs, there are incomplete reports on the use of *Chlorella vulgaris (C. vulgaris*). The possibility of this organism yielding a wide diversity of primary and secondary metabolites makes it a suitable candidate for the synthesis of nanoparticles. *Chlorella vulgaris*, a freshwater unicellular green microalga belonging to the family Chlorellaceae, is rich in proteins, lipids, carbohydrates, pigments, and fibre [13]. It contains essential bioactive nutrients such as carotenoids, chlorophyll, vitamins, and polyunsaturated fatty acids, contributing to its hepatoprotective, antioxidant, anti-inflammatory, anti-hypertensive, anti-hyperlipidemic, and antidiabetic effects [14].

Biogenic SeNPs represent a promising candidate for advanced clinical research due to their low toxicity and excellent biocompatibility; however, studies exploring their clinical applications remain limited [15]. Currently, Biogenic selenium nanoparticle–based therapeutic approaches are still in their early developmental phases but are moving toward clinical trial readiness. In this study, we investigated the potential therapeutic value of biogenic selenium nanoparticles produced with Chlorella vulgaris (SeNPs-CV) under conditions of severe metabolic stress using a high-dose BPA model.

## Materials and methods

### Chemicals and assay kits

Bisphenol A, with 99.5% purity, is sourced from Sigma–Aldrich Co. (St. Louis, MO, USA). Sodium selenite and selenium dioxide (Na_2_SeO_3_) were also purchased from Sigma-Aldrich, Egypt. *Chlorella vulgaris* algal culture was purchased from Dokki National Research Center. Commercial Reduced Glutathione (GSH) Assay Kit (Colorimetric) (Catalogue #K464-100), malondialdehyde (MDA) Assay Kit (Colorimetric) (Catalog # K739-100), and superoxide dismutase (SOD) assay kit (catalog # K335-100) were obtained from BioVision, Inc., Milpitas, USA. Additional kits used for lipid profile in rats included those for HDL ELISA kit (catalogue number: MBS266554, MyBioSource Co., USA), Total Cholesterol ELISA kit (catalogue number: K4436-100, BioVision Inc., USA), Triglyceride ELISA kit (catalogue number: EK720636, AFG Scientific Co., USA), and low-density lipoprotein (LDL) ELISA kit (catalogue number: MBS706180, MyBioSource Co., USA) were also supplied. Leptin ELISA kit (catalogue number: LS-F318, LifeSpan Biosciences, USA), adiponectin ELISA kit (catalogue number: E-EL-R3012, Elabscience, USA), glutathione peroxidase (GPx) ELISA kit (catalogue number: E1172Ra, Bioassay Technology Laboratory, China), TNF-α ELISA kit (catalogue number: 438204, BioLegend Inc., USA), and Rat Nuclear Factor Kappa B p65 (NF-κB -p65) ELISA Kit (catalogue number: E-EL-R0674, Elabscience, USA) were provided. Colorimetric activity assay kits for alanine aminotransferase (ALT) (cat no: MET-5123) and aspartate aminotransferase (AST) (cat no: MET-5127) were provided by Cell Biolabs, Inc., Creating Solutions for Life Science Research, Cairo branch, Egypt. Kits for albumin were provided by Diamond Co., Egypt. The remaining reagents were all commercially available and of analytical quality.

### Methods

#### Preparation of SeNPs

Selenium nanoparticles (SeNPs) were synthesized through physical and biological methods.

##### SeNPs preparation by physical method

Selenium nanoparticles (SeNPs) were produced at Beni-Suef University’s Nanotechnology Laboratory of Postgraduate Studies for Advanced Sciences Faculty, using a standard synthesis method (high-energy ball mill; HEBM), which is a top-down physical approach to nanomaterial fabrication [16]. It relies on mechanical forces to break bulk material into nanoparticles.

##### SeNPs preparation by biological method (biogenic synthesis of SeNPs)

*Chlorella vulgaris* was obtained from the Algal Biotechnology Unit’s culture collection (National Research Centre, Dokki, Giza, Egypt). The microalgal biomass was initially shade-dried at room temperature (25 ± 2 °C) for 48 h, then further dried in a convection oven at 60 °C for 15 min. The dried material was finely ground using an electric mixer. A 50 g portion of the powdered *Chlorella vulgaris* was suspended in 500 mL of deionized water and thoroughly mixed by magnetic stirring at 500 rpm for 30 min. This mixture was centrifuged for 10 min at 10,000 rpm, and then the supernatant was collected to serve as the reducing and capping agent. In a 1 L flask, 500 mg of sodium selenite (Na₂SeO₃, ≥ 95% purity, Sigma-Aldrich, Cat. No. S5261, USA) was dissolved in 50 mL of deionized water and subsequently added to 550 mL of the freshly prepared C. vulgaris aqueous extract. The precursor concentration was 0.91 mg/mL (~ 4.6 mM sodium selenite), and then the solution was agitated continuously at room temperature for twenty-four hours. A colour change from colourless/pale green to brick-red was observed within 4–6 h, intensifying over the full 24 h period, indicating the synthesis of selenium in nanoparticle form. The reduction of metal salt into metal nanoparticles can occur by the actions of carbohydrates and proteins of the algal extract. The formed nanoparticles were then separated by centrifugation for 10 min at 10,000 rpm and frequently washed with deionized water to eliminate residual selenium ions and organic byproducts [11]. Synthesis yield is about ~ 180–210 mg of biogenic selenium nanoparticles. Stock concentration of re-dispersed Biogenic SeNPs-CV in 500 mL deionized water is ~ 0.36–0.42 mg/mL. The same conditions were used to create three separate synthesis batches. The mean hydrodynamic diameter (by DLS) had a relative standard deviation (RSD) of less than 12%, indicating satisfactory batch-to-batch repeatability for the production of biogenic nanoparticles.

#### SeNPs and SeNPs-CV characterization

##### Transmission electron microscope (TEM)

The produced SeNPs and biogenic SeNPs-CV were characterized by Transmission Electron Microscopy (TEM) (model JEM-2100, JEOL Ltd., Tokyo, Japan) [17] to show the shape and size of nanoparticles. A drop of the aqueous selenium nanoparticle sample was placed onto a carbon-coated copper grid and then left to air dry completely at room temperature for one hour. Then the sample was examined under a microscope, and clear images were captured at various magnifications.

##### Dynamic light scattering (DLS) analysis

DLS characterization for SeNPs and the biosynthesized selenium nanoparticles is carried out by a particle size analyzer manufactured by Malvern Panalytical NanoSight NS500 instrument [18].

##### Zeta potential analysis

The SeNPs and biosynthesized SeNPs-CV were characterized for their physicochemical properties using a ZetaSizer Nano ZS particle analyzer (v7.11, Malvern Instruments, UK) [19] to determine particle size distribution and Zeta potential.

##### UV–visible spectroscopic analysis

Using a double-beam UV-Vis spectrophotometer and deionized water as the reference, the UV-visible absorption spectra of the biosynthesized SeNPs-CV colloidal solution were captured in the 200–800 nm wavelength range. Quartz cuvettes with a 1 cm route length were used for the measurements, which were carried out at room temperature. Additionally, the aqueous extract of *C. vulgaris* was assessed as a blank control for comparison.

##### X-ray diffraction (XRD) analysis

The X-ray diffraction (XRD) analysis for both SeNPs and the biosynthesized selenium nanoparticles was conducted using a D8-Find diffractometer (Bruker, Madison, WI, USA) with Cu Kα radiation (λ = 1.5418 Å) under operating conditions of 40 kV and 40 mA, with a step filter of 0.01° [20].

### Animals

40 adult male albino rats (weight 160–180 g) were purchased from the Animal Research House at NUB farm (Beni-Suef, Egypt). Rats were kept in a ventilated room under optimum conditions. Free access to water and a standard rodent diet were introduced. All experimental measures were approved by the Institutional Animal Care and Use Committee of Beni-Suef University (approval number 021–209) and followed the ARRIVE (Animal Research: Reporting of In-Vivo Experiments) guidelines (https://arriveguidelines.org).

### Experiment design

After seven days of adaptation, the rats were arbitrarily allocated into 4 groups (*n* = 10 per group) as follows:

Control group: rats orally administered corn oil (2 ml/kg/day) as a vehicle for BPA.

BPA group: rats were orally administered BPA in corn oil (150 mg/kg body weight) (5 days/week) for 45 days via gastric tube. The dose was equal to 1/20 of the median lethal dose [21]. This dosage was selected based on earlier toxicological investigations that consistently induced metabolic disorders and oxidative stress in rats throughout the study, despite exceeding typical environmental exposure.

SeNPs group: rats orally received Selenium nanoparticles (SeNPs) in distilled water (2 mg/kg body weight) [22] (5 days/week) for 45 days by gastric tube and were administered BPA with the same dose as in BPA group.

SeNPs-CV group: rats were gavaged SeNPs-CV (2 mg/kg body weight) [22] (5days/week) for 45 days by gastric tube, and BPA with the same dose as in the BPA group.

Rats were observed weekly for behavioural changes, signs of toxicity, mortality, and body weight changes.

### Sampling and tissue preparations

All animals were fasted overnight after the last dose of different treatments, and blood samples were drawn from the retro-orbital sinus. They were centrifuged (10 min at 3000 r/min within 30 min to prevent auto-glycolysis) for serum separation. The collected sera were preserved at – 20 °C until the time of use. Animals were humanely exposed to cervical dislocation after anesthesia by IP injection of 0.1 ml / 100 kg /rat from the mixture of ketamine (90 mg/kg) and xylazine (5 mg/kg). After collecting and washing with physiological saline, the livers were separated into three portions. A piece was fixed in 10% neutral-buffered formalin for histopathology. Another fraction (0.5 g) was homogenized in 5 mL of phosphate-buffered saline using a Teflon homogenizer and centrifuged at 3000 rpm for 10 min at 4 °C. The resulting supernatants were stored at − 20 °C until oxidative and antioxidant parameters were analyzed. The final part of liver tissue was preserved at − 80 °C for molecular analyses.

### Determination of body weight gain %

Each rat’s body weight was recorded once a week during the trial. The body weight gain percentage (BWG %) was calculated following Chapman et al. [23]

BWG % = [(Final body weight − Initial body weight) / Initial body weight] × 100.

### Coefficient weights of liver

All rats’ body weights were measured before they were sacrificed. After weighing the body and liver, the liver-to-body weight coefficients were calculated using the organ weight (mg) to animal body weight (g) ratio.

### Biochemical assays

#### Determination of lipid profile in serum

For serum lipid profile analysis in all rat groups, Total cholesterol, Triacylglycerols, HDL, and LDL levels were estimated using commercial kits following the manufacturer’s guidelines based on sandwich ELISA [24]. Calculation of very low-density lipoprotein (VLDL) and LDL cholesterol concentrations was conducted employing Friedewald’s equation [25]:

VLDL cholesterol = Triglyceride/5

LDL cholesterol = Total cholesterol - (HDL + VLDL cholesterol)

#### Determination of hepatic Redox markers

Lipid Peroxidation (MDA) was assayed colorimetrically in liver homogenate by TBARS assay based on Albro et al. [26]. Reduced Glutathione (GSH) levels were assayed in homogenate by an enzymatic recycling assay originally based on the method by Tietze [27]. SOD activity was assessed using the method of Sun et al. [28], and Glutathione peroxidase (GPx) was estimated in liver homogenate by ELISA assay.

#### Determination of liver function tests

The albumin serum level was measured following the bromocresol green method [29]. ALT and AST activities were measured in serum as described by Steven [30].

#### Determination of Hepatic lipogenic genes (SREBP1, ACC1, FAS, miRNA-122) by Quantitative RT-PCR analysis

The mRNA expression levels were analyzed using real-time quantitative polymerase chain reaction (qPCR). The Direct-zol RNA Miniprep Plus kit (Cat # R2072, Zymo Research, USA) was used to extract total RNA from liver tissues. The extracted RNA’s concentration and purity were measured using a Beckman dual spectrophotometer (USA). Total RNA was converted into complementary DNA (cDNA) using the Invitrogen™ SuperScript™ IV One-Step RT-PCR kit (Cat# 12594100, Thermo Fisher Scientific, Waltham, MA, USA). Real-time qPCR amplification and analysis were performed on an Applied Biosystem with software version 3.1 (StepOne ™, USA). Table 1 lists the primer sequences for the genes that were examined. SYBR Green Master Mix (Step One Applied Biosystem, Foster City, USA) was present in the reaction. Glyceraldehyde 3-phosphate dehydrogenase (GAPDH) expression as a housekeeping gene was used to standardize the relative expression values of the target genes (SREBP1, ACC1, FAS, and miRNA-122). The Cycle threshold (Ct) values of the evaluated genes in comparison to the matching internal control are included in the PCR data sheet. All these steps were completed in line with the method provided by Livak and Schmittgen [31]. The data were expressed in and calculated according to the delta-delta Ct (ΔΔCt). Relative quantification (RQ) was calculated for each gene by calculating 2^−∆∆Ct^. Every sample underwent two analyses [32].

**Table 1:** Primers sequence used for real-time PCR

	Forward sequence	Reverse sequence	Gene accession number
SREBP1	ACA AGA TTG TGG AGC TCA AG	TGC GCA AGA CAG CAG ATT TA	NM_001276708.1
ACC1	TGAAGGGCTACCTCTAATG	TCACAACCCAAGAACCAC	NM_022193.2
FAS	TCCCAGGTCTTGCCGTGC	GCGGATGCCTAGGATGTGTGC	NM_017332.2
GAPDH	GGACTCATGACCACAGTCCAT	CAGGGATGATGTTCTGGAGAG	NM_000194.2
miRNA-122	UGUGACAAUGGUGUUUGUG (Stem loop sequence)		MIMAT0000827
U6	GTGCTCGCTTCGGCAGCACATATACTAAAATTGGAACGATACAGAGAAGATTAGCATGGCCCCTGCGCAAGGATGACACGCAAATTCGTGAAGCGTTCCATATTTT		XR_007492463.1

#### Determination of serum Inflammatory modulators (Adiponectin, Leptin, TNF-α, and NF-κB-p65)

Adiponectin, Leptin, TNF-α, and NF-κB-p65 levels were estimated in the serum of rats by a sandwich ELISA [24], using commercial assay kits following the instructions of the manufacturer.

### Histopathological investigation

The liver tissues were fixed for 48 h with 10% neutral-buffered formalin. Following the procedure outlined by Bancroft and Gamble [33], they were then cleaned with water, dehydrated in graded ethyl alcohol, clarified in xylene, and fixed in paraffin wax at 70 °C. Five µm-thick tissue was stained with hematoxylin and eosin (H & E). An optical microscope was used to examine slides to assess tissue changes. Five animals per group were included in the histopathological examination and scored, and for each slide, five sections were read, with five microscopic fields examined in each section. Hepatic tissue damage was semi-quantitatively scored on a scale of 0 = no change; 1 ≤ 25% tissue damage; 2 = 26–50% tissue damage; 3 = 51–75% tissue damage; and 4 = 76–100% tissue damage. All treatment groups underwent blind histopathological examinations.

###  Statistical analysis

GraphPad Prism 5 (Version 7.0, San Diego, CA, USA) was used for statistical analysis. One-way analysis of variance (ANOVA) was done, followed by Tukey’s multiple comparisons test for equal sample size. Exact adjusted p-values are reported where applicable. Statistical significance was considered at *p* < 0.05.

## Results

Throughout the 45-day study period, the animals were closely monitored, and no signs of toxicity, such as abnormal behaviour, weight loss, or mortality, were observed, confirming the safety and absence of inherent toxicity of SeNPs and SeNP-CV.

### Characterization of SeNPs

#### TEM analysis

**Fig. 1:**
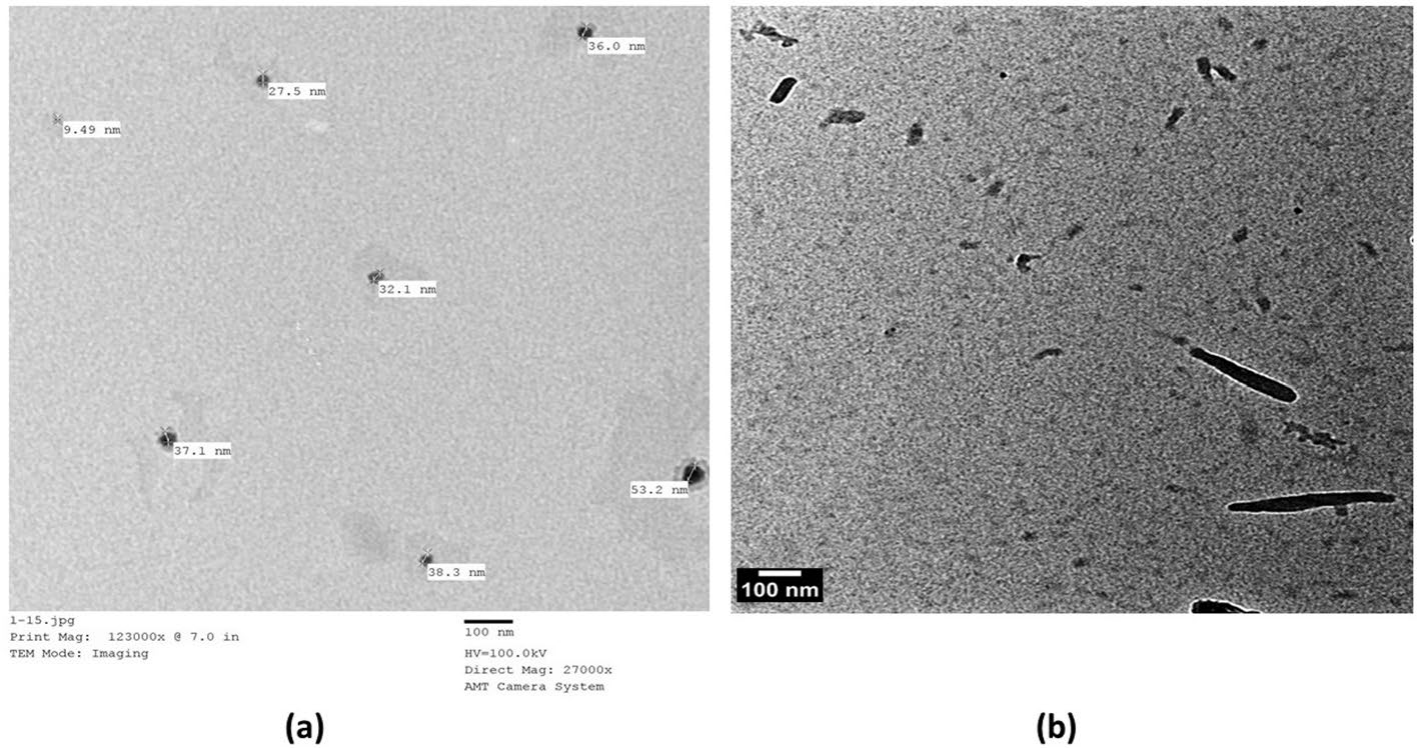
TEM of selenium nanoparticles showing: **a** spherical particles of SeNPs. and **b** rod-like elongated particles of SeNPs-CV, indicating a successful preparation

Using TEM, the morphology of physically prepared Se-NPs was studied as shown in Fig. 1a which revealed uniformly distributed spherical particles with no agglomeration. The nanoparticle diameters ranged from 9 to 53 nm. The TEM examinations of the produced Se-NPs showed that the Se nanoparticles have higher aspect ratios. Moreover, no additional morphologies were seen, and the measured morphologies of the generated Se nanostructure were clearly nanoparticles. TEM image of biogenic SeNPs was shown in Fig. 1b. Results revealed that CV-mediated synthesis produced well-dispersed, predominantly rod-like elongated nanoparticles (50–100 nm) with uniform morphology and size distribution (Fig. 1b), confirming successful green synthesis.

#### Zeta potential analysis

**Fig. 2:**
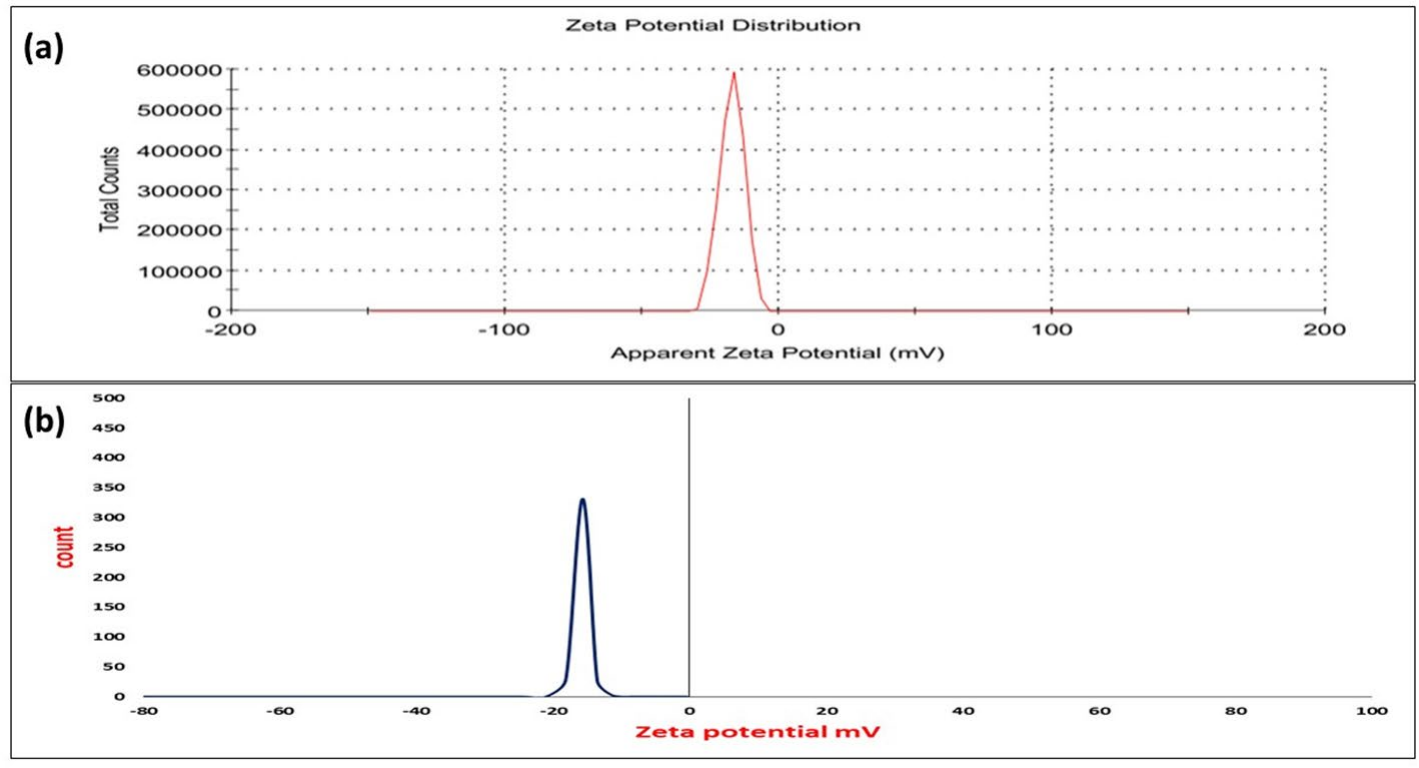
Zeta potential of SeNPs (a), and biogenic SeNPs-CV (b)

The Zeta potential values of SeNPs and the prepared biosynthesized SeNPs-CV revealed a strongly negative zeta potential of – 17.1 mV (Fig. 2a) and − 15 mV (Fig. 2b), respectively, confirming moderate colloidal stability attributed to high electrostatic repulsion. Overall, these findings confirm the successful synthesis of stable, nano-sized SeNPs. Triplicate analyses of each sample were performed.

#### Dynamic light scattering (DLS)

**Fig. 3:**
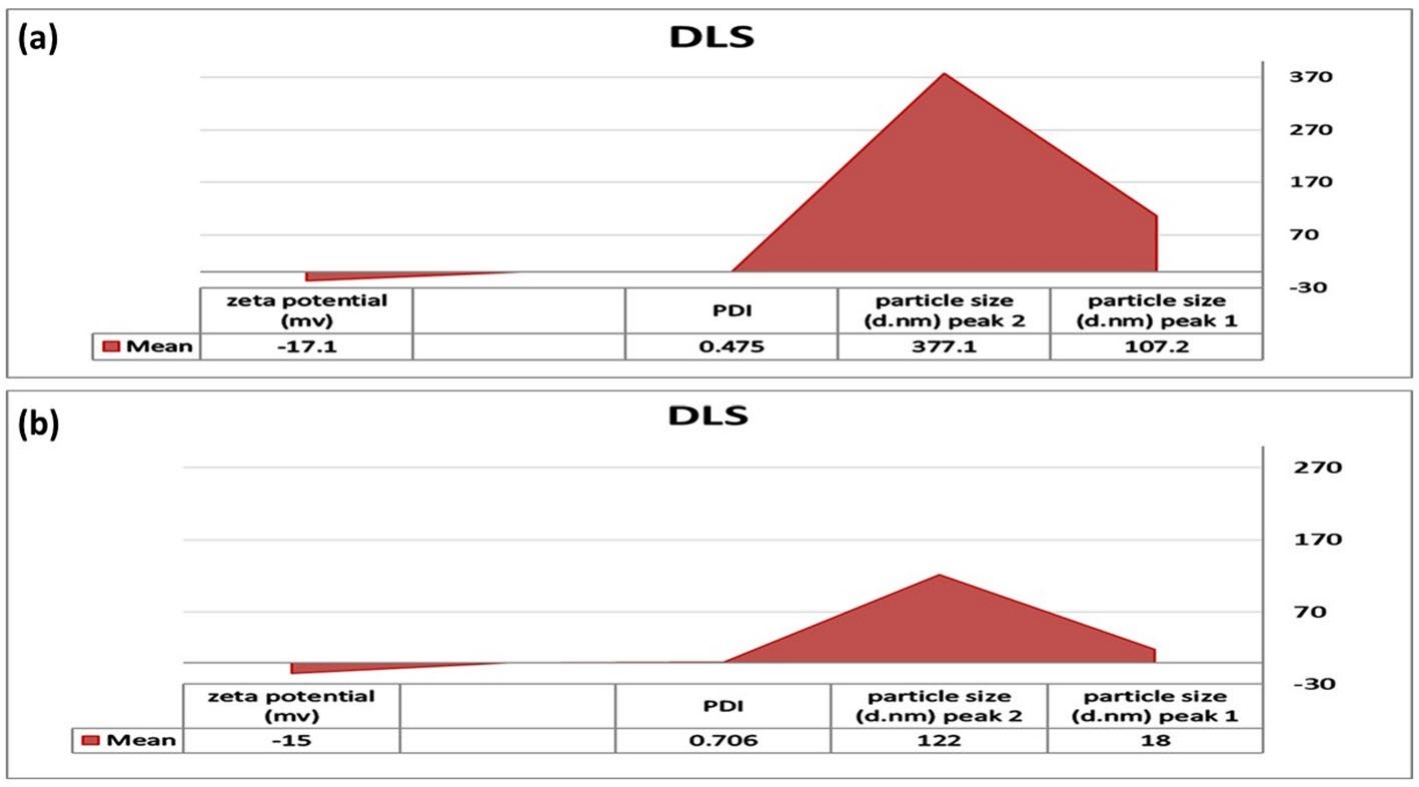
(a) DLS of SeNPs, peak 1= 107.2 nm, peak 2= 377.1 nm. (b) DLS of SeNPs-CV, peak 1= 18nm, peak 2= 122nm.

Dynamic light scattering (DLS) analysis was conducted to measure the hydrodynamic diameter, which includes the hydration layer, capping agents (e.g., proteins or polymers), and any degree of reversible agglomeration in the liquid phase. DLS analysis revealed a bimodal particle size distribution for the particles (Fig. 3). Physically and biosynthesized Se-NP samples were analyzed by DLS, as shown in Fig. 3. The results showed a particle size diameter of SeNPs ranging from 107.2 to 377.1 nm (Fig. 3a). The size of biogenic SeNPs-CV was 18 to 122 nm (Fig. 3b). However, the particle sizes measured by DLS were slightly larger than those observed with TEM, likely due to the hydrodynamic layer of water molecules or capping agents surrounding the nanoparticles or capping agents [34]. The Polydispersity Index (PDI) values of 0.475 for SeNPs and 0.706 for biogenic SeNPs-CV further explain the broad, bimodal nature of the size distribution.

##### UV–Vis spectroscopic analysis of biogenic SeNPs-CV

**Fig. 4:**
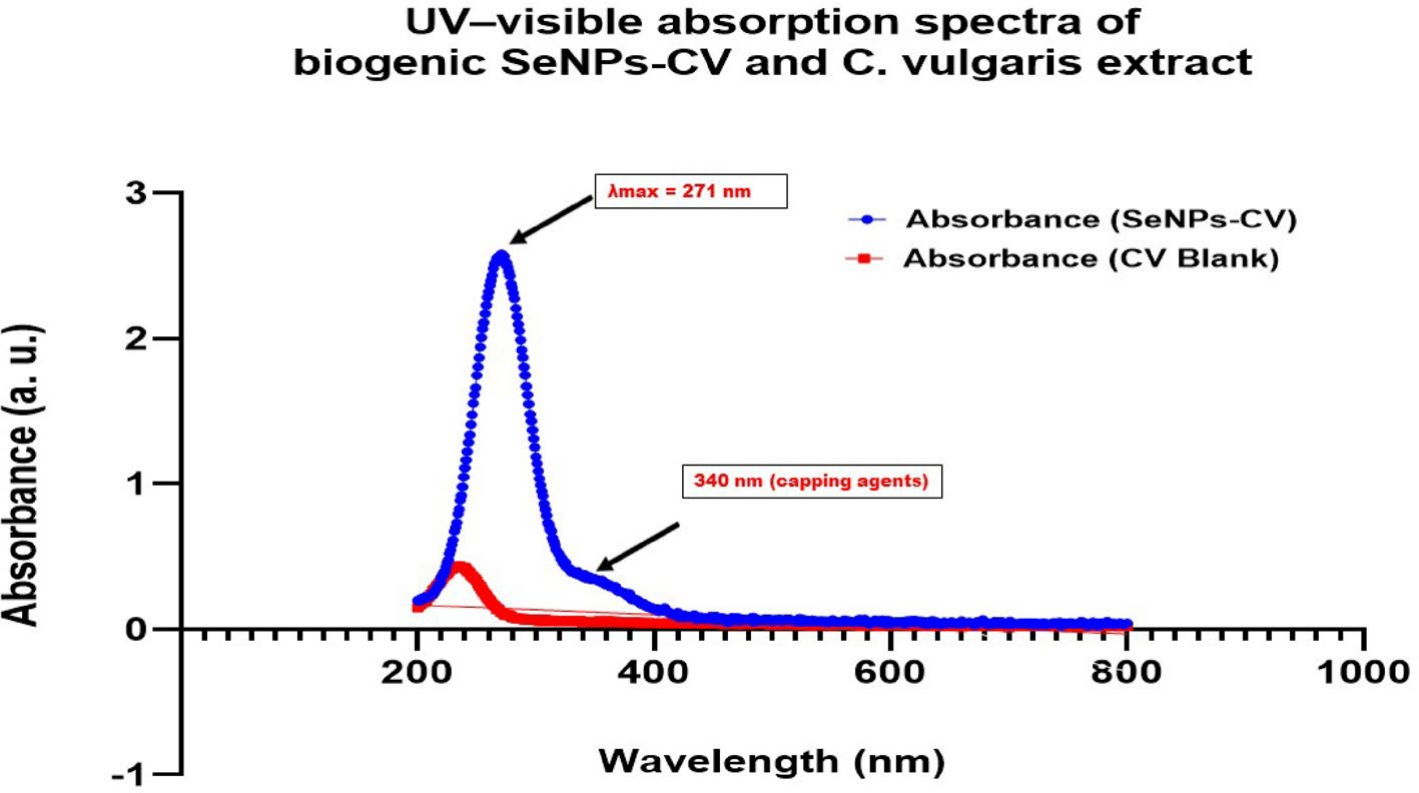
UV–visible absorption spectra of *C. vulgaris* extract and biogenic SeNPs-CV: The whole spectrum range (200–800 nm) displays the distinctive SPR peak at λmax = 271 nm. The SPR band is shown by the darkened area (220–500 nm), with the organic capping agents responsible for the major peak and shoulder at around 340 nm. Conditions: DI water reference, quartz cuvette, and 1 cm route length

UV-visible spectroscopy tracked the bio reduction process and verified that SeNPs-CV had formed successfully. Within the usual 250–300 nm range observed for biogenic SeNPs, the spectra of the biogenic SeNPs-CV colloidal solution had a clear surface plasmon resonance (SPR) peak at λmax = 271 nm (Fig. 4), which is typical of elemental selenium nanoparticles. The narrow full-width at half maximum (FWHM) of the SPR peak (54 nm) indicates moderate size uniformity, whereas a weak shoulder (340 nm) is attributed to *C. vulgaris* biomolecules functioning as capping agents. SeNP formation was confirmed by the extract’s lack of an SPR band. The brick-red color and the DLS, zeta potential, TEM, and XRD data are compatible with selective selenite reduction, which is supported by the lack of other visible peaks.

##### X-ray diffraction (XRD) evaluation

**Fig. 5:**
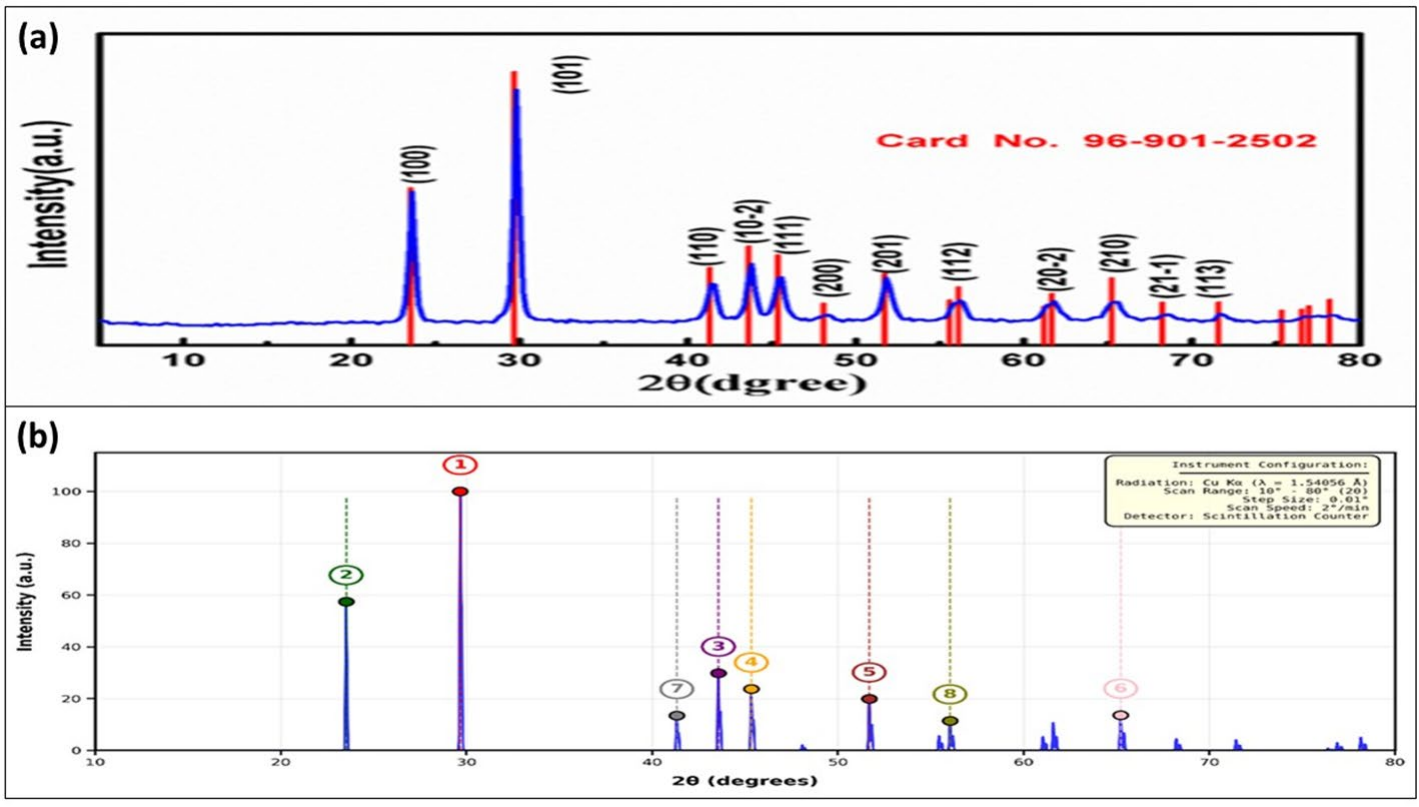
XRD pattern of SeNPs (**a**) and SeNPs-CV (**b**). **a** X-ray diffraction pattern of SeNPs arranged at 2θ of 23.6, 30.07, 41.57, 44.10, 45.80o, 51.7o, 50.19o, 61.58o, 64.97, 68.60, and 71.80 corresponded to the crystal planes of (100), (101), (110), (10−2), (201), (20−2), (201), (21−1) and (113) of (JCPDS card No. 096-901-2502) standard. The Se NPs having a hexagonal structure were successfully formed, and the lattice constants were a = 4.36 Å and c = 4.36 Å as per (JCPDS card No. 096-901-2502) standard. The peak intensities of (100) and (101) planes were enhanced, suggesting that the Se NPs have been favoring to grow along the (202) direction. **b** X-ray diffraction pattern of biosynthesized selenium nanoparticles (SeNPs-CV) demonstrating crystalline phase characteristics. Experimental conditions: Cu Kα radiation (λ = 1.54056 Å), angular range 2θ = 10–80°, step size 0.01°, scan speed 2°/min. Eight indexed peaks (1–8) correspond to characteristic reflections of the hexagonal selenium crystal structure. Peak intensities normalized to the maximum value (Peak 1 at 2θ = 29.66°). Detection system: scintillation counter, ambient laboratory conditions

By using X-ray diffraction (XRD) analysis, the produced nanoparticles’ composition and structure were determined, as depicted in Fig. 5 within the 2θ range of 10–80°. The absence of contamination peaks, along with the presence of only sharp and narrow peaks, suggests that the nanoparticles exhibit high purity and are well-crystallized Se NPs. The XRD pattern, as illustrated in Fig. 5a was used to study the crystallinity of selenium nanoparticles. The selenium peaks arranged at 2*θ* of 23.6, 30.07, 41.57, 44.10, 45.80o, 51.7o, 50.19o, 61.58o, 64.97, 68.60, and 71.80 paralleled to the crystal planes of (100), (101), (110), (10–2), (201), (20 − 2), (201), (21−1) and (113) of (JCPDS card No. 096-901-2502) standard. The hexagonal structure of the Se NPs was effectively generated, and the lattice constants were a = 4.36 Å and c = 4.36 Å. The increased peak intensities of the (100) and (101) planes indicate that the (202) direction has been selected for the growth of Se NPs.

The diffractogram of biogenic SeNPs-CV (Fig. 5b) displays eight sharp and well-resolved peaks at 2θ values of 29.66°, 23.51°, 43.56°, 45.33°, 51.68°, 65.22°, 41.32°, and 56.03°, corresponding well with the standard reference pattern of hexagonal selenium (JCPDS card No. 06-0362). The dominant reflection at 29.66°, indexed to the (101) plane, confirms the formation of the hexagonal phase. Calculated d-spacing values and peak indexing further validate that the biosynthesized Se NPs adopt the trigonal/hexagonal crystal structure (space group P3₁21). The uniform peak broadening (FWHM = 0.50°) indicates a monodisperse crystallite size distribution with minimal lattice strain.

### Effect of SeNPs-CV on liver weight, coefficients, and body weight gain %

BWG% was the highest in the BPA group after 30 and 45 days of the experiment. On the other hand, the SeNPs and SeNPs-CV groups preserved normal weight during the investigation period. The biogenic Selenium nanoparticles significantly reduced BWG% after 15 (*p* = 0.01 and 30 (*P* = 0.04) days in comparison with the SeNPs group (Table 2). As depicted in Table 2, liver weight and coefficients were pointedly increased (*p* < 0.0001) in the BPA group versus the control one. These changes were expressively restored in other co-treated groups with either SeNPs or SeNPs-CV as compared to BPA group (*P* = 0.01).

**Table 2:** Changes in Body weight gain %, liver weights, and liver coefficients in different groups

Groups	BWG% after 15 days	BWG% after 30 days	BWG% after 45 days	Liver weight (g)	Liver coefficients (mg/g)
Control	20.51 ± 1.91 ^ab^	47.56 ± 1.84 ^a^	77.07 ± 2.51 ^ab^	7.89 ± 0.73^a^	33.16 ± 2.99^a^
BPA	27.17 ± 3.14 ^b^	73.48 ± 8.03 ^b^	91.4 ± 3.37 ^b^	13.8 ± 0.73^b^	55.72 ± 4.61^b^
SeNPs + BPA	28.73 ± 2.53 ^b^	71.38 ± 7.19 ^b^	77.37 ± 3.68 ^ab^	9.43 ± 0.65^a^	34.78 ± 2.65^a^
SeNPs-CV + BPA	16.85 ± 1.88 ^a^	47.95 ± 4.03 ^a^	71.48 ± 6.09 ^a^	9.29 ± 0.42^a^	32.66 ± 2.04^a^
*P* value	0.0058	0.0034	0.0157	< 0.0001	< 0.0001

Values are represented as mean ± standard error (*n* = 7). Different superscript letters (a, b) indicate significant differences between groups within the same column at the reported *p values*. Body weight gain percentage (BWG %)

### Effect of BPA and SeNPs-CV on lipid profile

Total cholesterol, TG, VLDL, LDL levels, and AI were pointedly higher (*p* < 0.0001) in BPA group as compared to the control. Nevertheless, there was a notable diminution in HDL levels (*p* = 0.0017) in the BPA group versus the control one (Table 3). CO-treatment with SeNPs or SeNPs-CV revealed an improved outcome by the modulation of the levels. Although no statistically significant differences were observed between the SeNPs and SeNPs-CV-treated groups, the biogenic selenium nanoparticles modestly improved the measured outcomes compared with SeNPs and exhibited a trend toward better efficacy across certain evaluated parameters.

**Table 3:** Changes in lipid profile in different studied groups

Groups	TG (nmol/l)	T. Cholesterol ( nmol/l )	VLDL ( nmol/l )	HDL nmol/l	LDL ( nmol/l )	AI
Control	1.123 ± 0.141 ^a^	2.283 ± 0.188 ^a^	0.225 ± 0.028 ^a^	1.158 ± 0.079^a^	0.900 ± 0.177^a^	0.994 ± 0.161^a^
BPA	3.293 ± 0.239 ^b^	5.48 ± 0.243 ^b^	0.659 ± 0.048 ^b^	0.819 ± 0.055^b^	4.001 ± 0.296^b^	5.859 ± 0.730 ^b^
SeNPs + BPA	2.744 ± 0.165 ^bc^	3.377 ± 0.165 ^c^	0.549 ± 0.033^bc^	0.983 ± 0.033^ab^	1.845 ± 0.171^c^	2.468 ± 0.263 ^a^
SeNPs-CV + BPA	2.18 ± 0.15 ^c^	3.334 ± 0.114 ^c^	0.436 ± 0.029 ^c^	1.004 ± 0.027^ab^	1.894 ± 0.112^c^	2.327 ± 0.132^a^
*P* value	< 0.0001	< 0.0001	< 0.0001	0.0034	< 0.0001	< 0.0001

Values are presented as mean ± standard error (*n* = 5). Different superscript letters (a, b, c) indicate significant differences between groups within the same column at the reported *p values*. Triacylglycerols (TG), Total cholesterol (TC), Very Low-density lipoprotein (VLDL). High-density lipoprotein (HDL), Low-density lipoprotein (LDL), AI (Atherogenic index)

### Effect of BPA and SeNPs-CV on lipogenic genes in liver

**Fig. 6:**
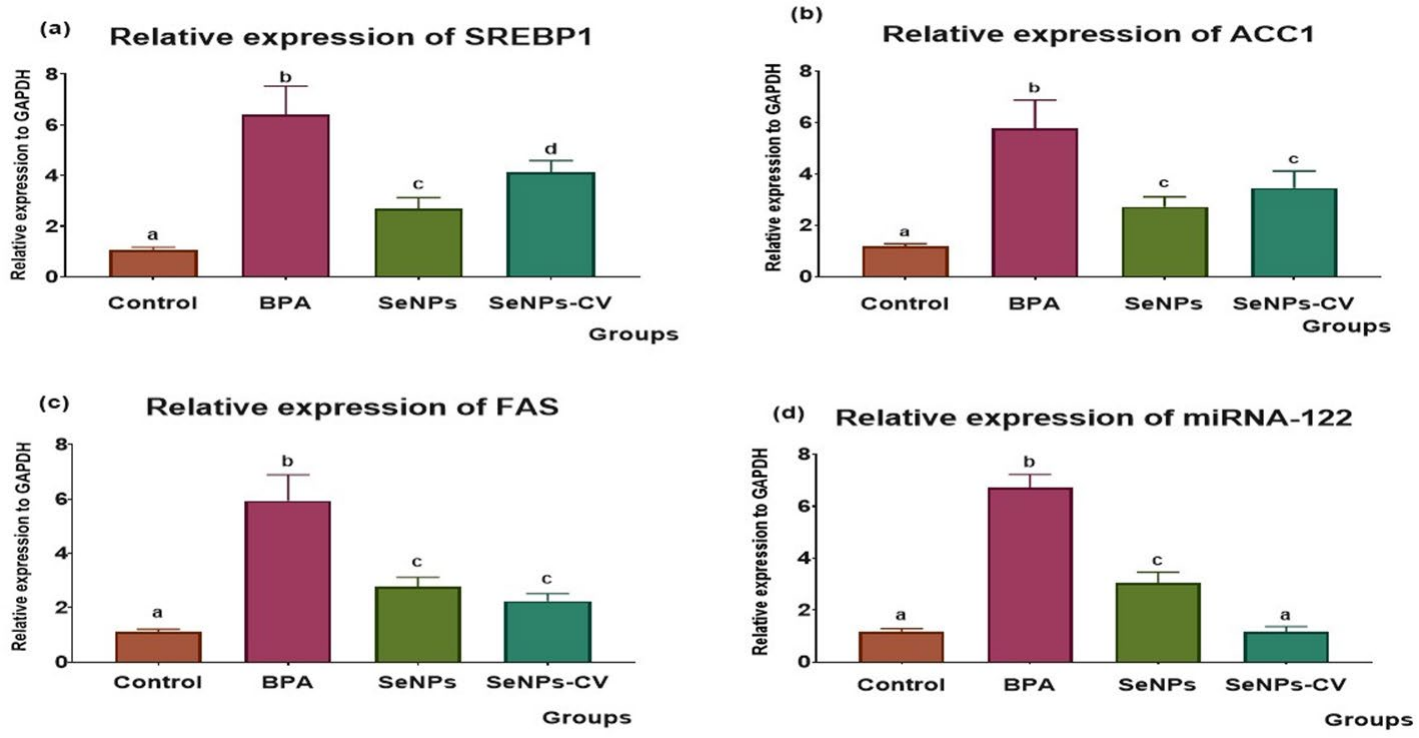
Changes in mRNA expression levels of (a) SREBP1, (b) ACC1, (c) FAS, and (d) miRNA-122 in hepatic tissues of different studied groups. Values are represented as mean ± standard error (*n* = 5). Different superscript letters (a, b, c) indicate significant differences between groups at *p* < 0.01. Sterol regulatory element binding protein-1 (SREBP1), fatty acid synthase (FAS), acetyl-CoA carboxylase (ACC1), microRNA-122 (miRNA-122)

The relative mRNA expressions of genes related to lipid metabolism (SREBP1, ACC1, FAS, and miRNA-122) in hepatic tissues in the different studied groups are presented in Fig. 6. The results displayed a significant upregulation (*p* < 0.0001) in SREBP1, ACC1, FAS, and miRNA-122 expression levels in BPA group compared to the control group. These values were expressively restored (*p* ≤ 0.01) in other co-treated groups with either SeNPs or SeNPs-CV as compared to BPA group. There are no variations in the values between SeNPs and SeNPs-CV-treated groups, except for miRNA-122 relative expressions, which are better in SeNPs-CV group than SeNPs group, demonstrating a trend toward better efficacy.

### Effect of BPA and SeNPs-CV on serum adipokines and inflammatory mediators

Serum leptin and TNF-α concentrations were meaningfully increased (*p* < 0.0001) in BPA group compared to those in the control group. While the concentrations of Adiponectin were meaningfully decreased (*p* < 0.0001) in BPA group versus the control group. NF-κB p65 level was measured as an indicator of total protein concentration. There were notable variations in total p65 levels between the BPA and the control groups (*P* < 0.0001). It should be emphasized, too, that this measurement does not discriminate between the activation status of NF-κB p65 and its total content.

**Fig. 7:**
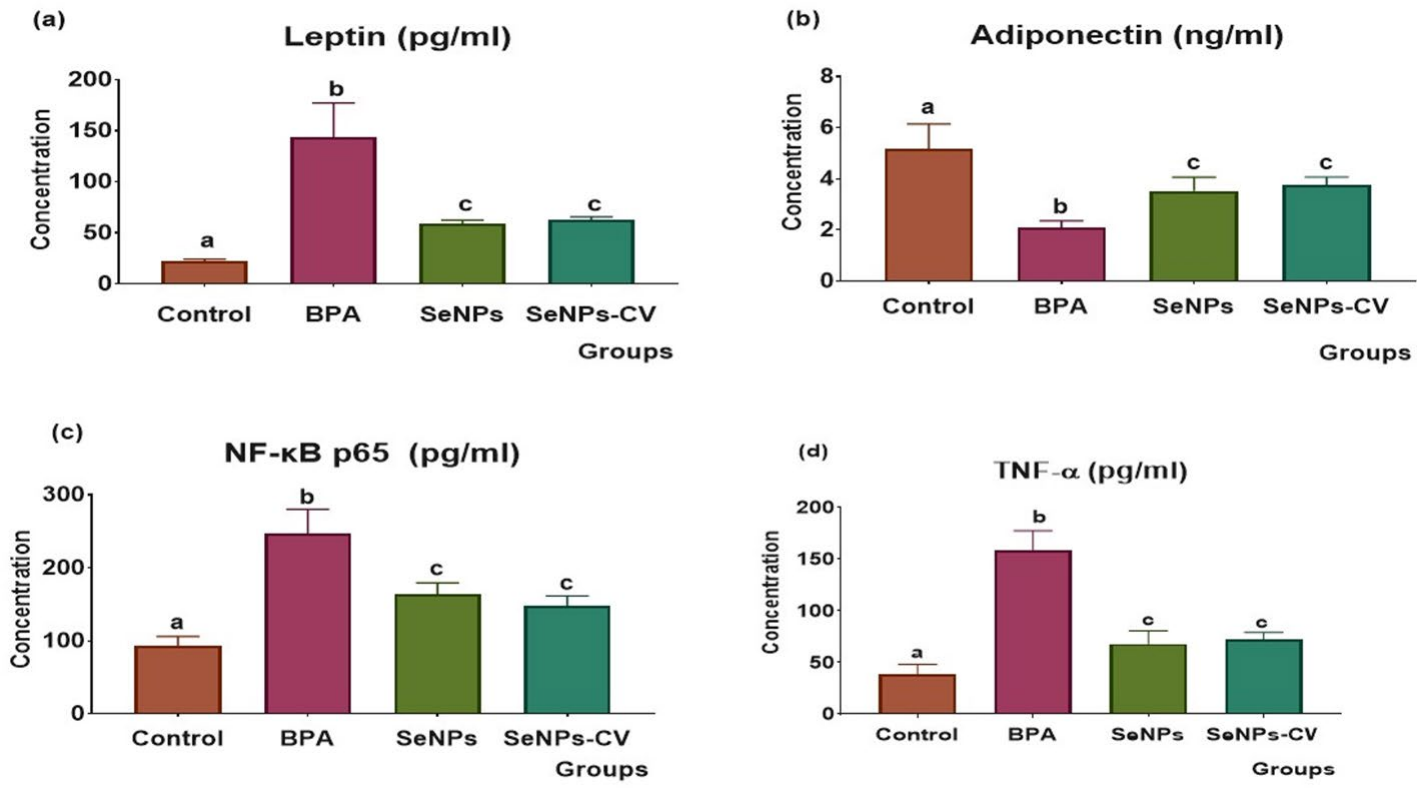
Changes in concentrations of (a) Leptin, (b) Adiponectin, (c) NF-κB p65, and (d) TNF-α in blood of different studied groups. Values are represented as mean ± standard error (*n* = 5). Different superscript letters (a, b, c) indicate significant differences between groups at *p* < 0.01. Nuclear factor kappa-B p65 (NF-κB p65), Tumor Necrosis Factor-alpha (TNF-α)

These values were expressively ameliorated (*p* < 0.01) in other treated groups (after co-administration of SeNPs or SeNPs-CV) matched to the BPA group. There are no noteworthy variations in the values between SeNPs and SeNPs-CV-treated groups (Fig. 7).

### Effect of BPA and SeNPs-CV on redox biomarkers in liver

**Fig. 8:**
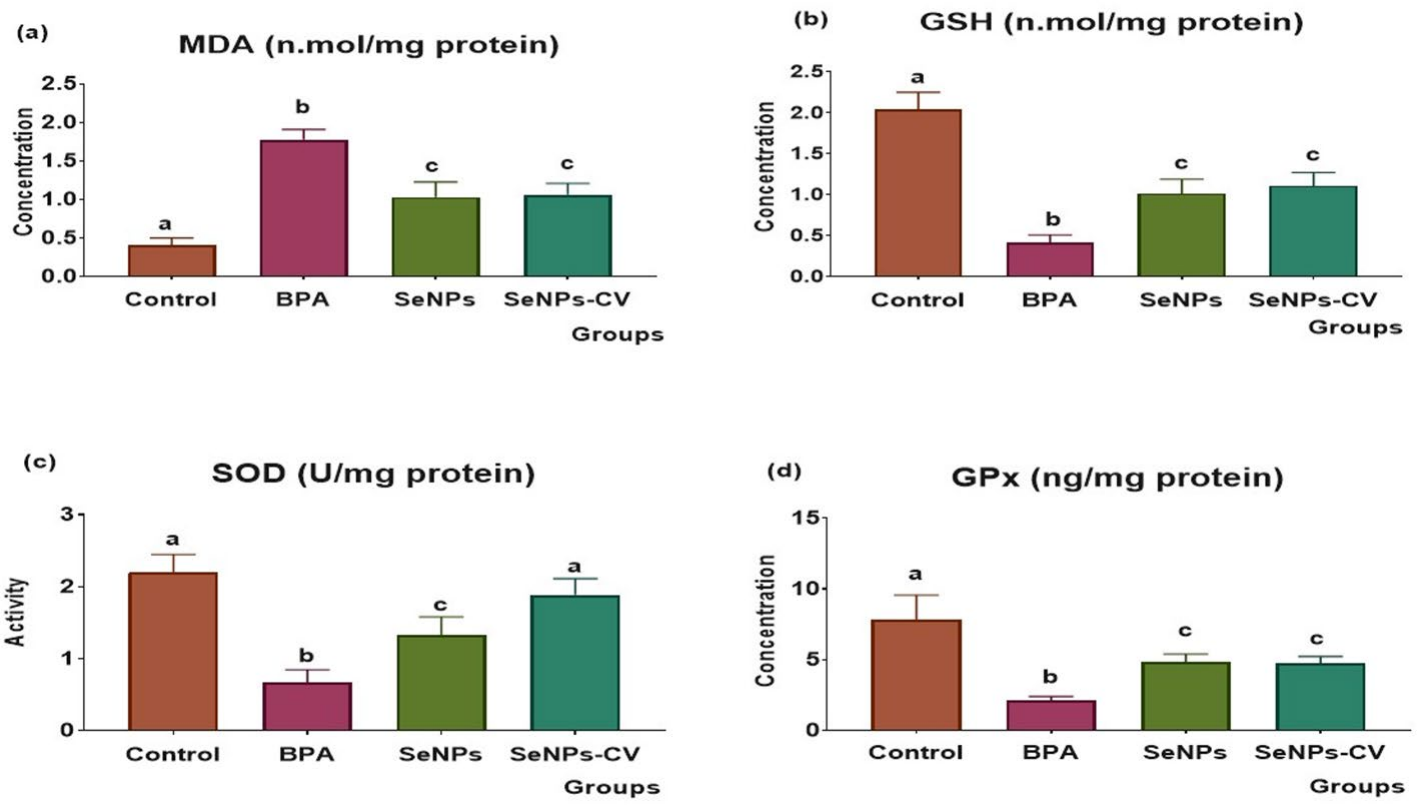
The concentrations of (a) GSH, (b) MDA, (c) SOD, and (d) GPx in hepatic tissues of different studied rat groups. Values are represented as mean ± standard error (*n* = 5). Different superscript letters (a, b, c) indicate significant differences between groups at *p* < 0.01. Reduced Glutathione (GSH), Malondialdehyde (MDA), Superoxide Dismutase (SOD), Glutathione peroxidase (GPx)

Results in Fig. 8 showed that the rats that received BPA had a noteworthy elevation (*p* < 0.0001) in the concentrations of hepatic MDA compared with those in the control group. While the levels of GSH, SOD and GPx activities in the liver significantly decreased (*p* < 0.0001). On the other hand, administration of SeNPs or SeNPs-CV showed significant amelioration (*p* < 0.01) of the values compared to BPA group, indicating an antioxidant effect of Selenium nanoparticles. There is no significant variation in the values between SeNPs and SeNPs-CV-treated groups, except for SOD values, which are better in SeNPs-CV than SeNPs group (*p* = 0.0068).

### Effect of BPA and SeNPs-CV on liver biomarkers

**Fig. 9:**
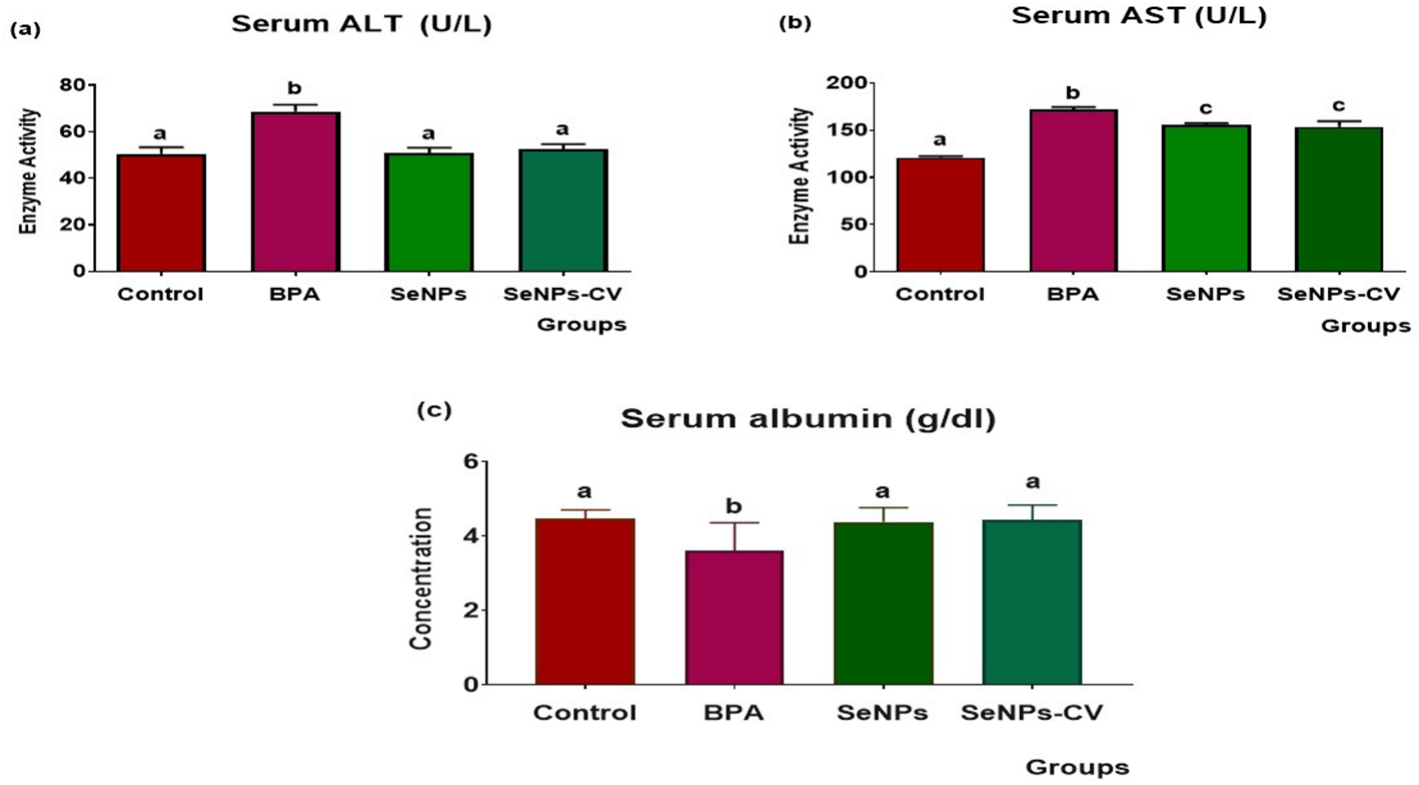
Changes in serum liver biomarkers (a) ALT, (b) AST, and (c) albumin in different studied groups. Values are represented as mean ± standard error (*n* = 7). Different superscript letters (a, b, c) indicate significant differences between groups at *p* < 0.0001 for ALT, AST and *p* = 0.0068 for albumin. Alanine Aminotransferase (ALT), Aspartate transaminase (AST)

The BPA group showed a significant increase in ALT and AST activities (*p* < 0.0001; *p* ≤ 0.01) and a significant decrease (*p* = 0.0141) in albumin levels compared with the control. Rats co-treated with SeNPs or SeNPs-CV demonstrated a significant improvement (*p* < 0.05) in these values compared to the BPA group. There was no significant difference between the SeNPs and SeNPs-CV-treated groups (Fig. 9).

### Histological examination

**Fig. 10:**
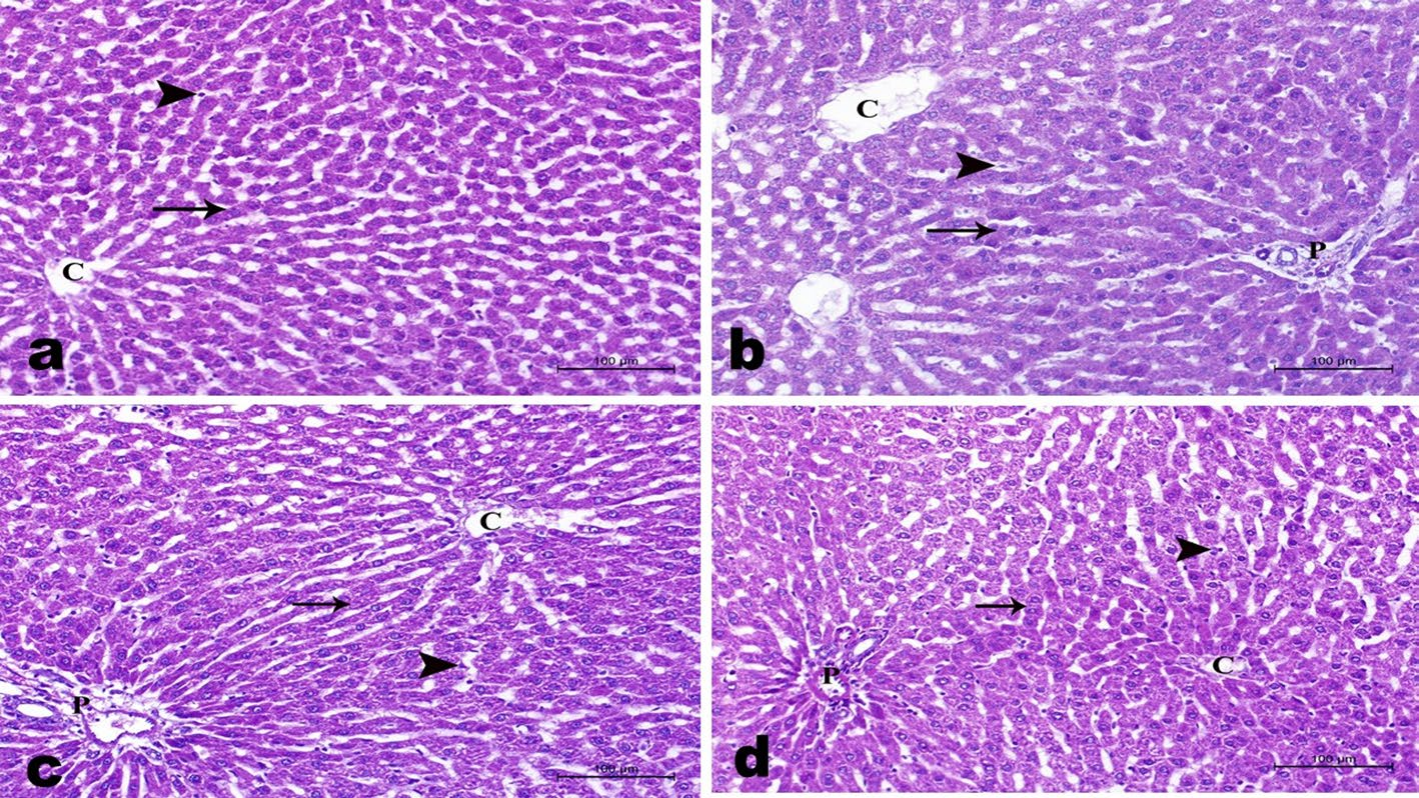
Photomicrograph of representative tissue samples from rats’ livers. **a** Control group showed normal arrangement of hepatic tissue with normal hepatocytes (arrow) and blood sinusoids (arrowhead) around normal central vein (C). **b** BPA group showed hydropic degeneration in hepatocytes (arrow) with wide sinusoids (arrowhead). The vessels of the central vein (C) and portal area (P) appeared dilated. **c** SeNPs group showed regaining normal hepatocytes (arrow) and blood sinusoids (arrowhead). The vessels of the central vein (C) and portal area (P) appeared almost normal. **d**: SeNPs-CV group showed normal hepatocytes (arrow) and blood sinusoids (arrowhead). The vessels of the central vein (C) and the portal area (P) appeared normal. (H&E Stain X400)

In the control group, the liver displayed normal hepatocytes with normal blood sinusoids around a normal central vein (Fig. 10a). In contrast, the BPA-exposed group showed hydropic degenerations in hepatocytes, wide sinusoids, with a dilated central vein and portal area (Fig. 10b). However, in the group treated with SeNPs or SeNPs-CV, the hepatocytes, blood sinusoids, central vein, and portal area appeared normal (Fig. 10c, d), indicating that SeNPs-CV may help restore hepatic structure and function damaged by BPA. The histopathological semi-quantitative scores of the tested groups were presented in Table 4. SeNPs-CV selectively improved the measured outcomes compared with SeNPs and demonstrated a trend toward better efficacy.

**Table 4:** Histopathological scoring in different studied groups

Group	Fibrosis	Congestion of central vein	Degeneration of hepatocytes	Sinusoidal dilatation	Portal vessels congestion	Van Kupffer cell hyperplasia
Control	0	0	0	0	0	0
BPA	2	3	3	3	3	3
SeNPs + BPA	1	1	1	1	1	2
SeNPs-CV + BPA	0	0	0	0	0	1

Histopathological scoring of hepatic tissue injury was scaled in degrees as follows: 0 = no change; 1 ≤ 25% tissue damage; 2 = 26–50%tissue damage; 3 = 51–75% tissue damage; 4 = 76–100% tissue damage

## Discussion

TEM images of biogenic SeNPs further validate their shape and nanoscale size [35]. The elongated rod form of SeNPs-CV may improve their ability as nanocarriers to move through tissues and organs more effectively than spherical SeNPs. Studies have indicated that rod-shaped particles can extend circulation time in the bloodstream and accumulate more in targets such as tumors, thereby enhancing therapeutic effectiveness [36].

The stability and hydrodynamic size of the SeNPs and SeNPs-CV were assessed using zeta potential and DLS analysis. The measured zeta potential of SeNPs and SeNPs-CV was − 17.1 mV and − 15 mV, respectively, indicating strong electrostatic repulsion and confirming that the nanoparticles are moderately stable and well-dispersed. The negative charge comes from polysaccharides and proteins in algae acting as reducing and capping agents [37]. DLS results showed particle sizes ranging from 107.2 to 377.1 nm and 18–122 nm for SeNPs and SeNPs-CV, respectively, which align with previously reported findings [38]. Consequently, the two peaks shown in the DLS profile are a direct result of the nanomaterial’s 2D anisotropic geometry rather than a sign that the particles are larger than the nanoscale threshold.

Biogenic nanoparticles are characterized by a wide and polydisperse system, as shown by the total PDI of 0.706. The production of smaller particles by primary nucleation and bigger ones by secondary growth or Ostwald ripening is probably the cause of this bimodality. The greater DLS values represent the hydrodynamic diameter, including the organic capping layer from *C. vulgaris*, while the lower values correspond with TEM observations. Furthermore, despite the observed polydispersity, the measured zeta potential (− 15 mV) provides sufficient colloidal stability through electrostatic repulsion.

The XRD pattern of the biosynthesized SeNPs shows sharp, well-defined peaks, confirming their crystalline and nanostructured nature [39]. Eight diffraction peaks are identified, indicating a polycrystalline material with a preferred orientation. Comparison with standard reference data reveals that the nanoparticles exhibit a pure hexagonal selenium phase, with the enhanced (101) plane indicating favoured directional growth. The broadening of the diffraction peaks further supports the nanoscale size of the particles. The formation of this stable hexagonal phase emphasizes the role of biological macromolecules as structure-directing agents, effectively lowering energy barriers for stable phase nucleation. This makes the biosynthesis pathway more thermodynamically efficient than conventional chemical methods.

In this study, the contribution of *Chlorella vulgaris* biomolecules to nanoparticle stabilization and biological activity is inferred from the green synthesis approach and supported by comparative characterization and biological results. However, no direct surface chemistry analyses were performed; therefore, the proposed capping and functional roles of these biomolecules should be regarded as a plausible interpretation rather than a confirmed mechanism.

Research on environmental exposure indicates that BPA can enter the food chain through plastic degradation, chemical leaching, and other mechanisms, resulting in prolonged low-dose exposure across the population [40]. Its xeno-estrogenic properties disrupt endocrine and metabolic pathways, altering glucose metabolism and raising the risk of developing diabetes, insulin resistance, and obesity [41].

*Chlorella vulgaris* is a green microalgal species rich in proteins, secondary metabolites, pigments, and peptides, which act as nano-bio factories. These bioactive compounds serve dual roles as reducing and capping mediators in the green preparation of selenium nanoparticles (Se-NPs). The use of algal biomass in this process enhances the nanoparticles with unique properties, owing to surface modifications mediated by diverse functional biomolecules [42]. Therefore, this study evaluated the therapeutic potential of green-synthesized selenium nanoparticles using *Chlorella vulgaris* (SeNPs-CV) in mitigating BPA-induced metabolic disturbances.

BPA effects on obesity were studied by the estimation of weight gain and liver weight of rats. Results indicated that BPA-exposed rats showed a significant increase in body weight gain percentage (BWG %) after 15, 30 and 45 days, along with higher liver weights and liver coefficients compared to controls (Table 2). This suggests that BPA contributes to obesity and metabolic disruption. These results align with earlier human studies, where higher urinary BPA levels correlated with increased waist circumference, indicating abdominal obesity [43]. However, our study revealed that co-treatment with SeNPs or SeNPs-CV significantly normalized BWG%, liver weights, and liver coefficients in BPA-exposed rats compared to the control group, with a more pronounced effect of biogenic SeNPs-CV than SeNPs, implying a protective effect. Previous studies reported that selenium promotes health and growth by facilitating the conversion of T4 to T3 and regulating the release of growth hormone, primarily through its role in 5’-deiodinase, a selenoprotein enzyme essential for thyroid hormone metabolism [44]. SeNPs may prevent weight gain by decreasing pathways like gluconeogenesis and proteolysis [45]. In a previous study, *Chlorella vulgaris* led to significant weight loss, although waist and hip measurements were unchanged [46]. This weight reduction may stem from the high carotenoid content in C. vulgaris, which inhibits lipid absorption and transport in the gastrointestinal tract, thus lowering plasma lipid levels and promoting weight loss [47].

In the current study, BPA-exposed rats exhibited a noteworthy dyslipidemia marked by higher levels of TC, TG, LDL, VLDL, and atherogenic index (AI), and decreased levels of HDL (Table 3). These results align with previous studies. For example, Hassani et al. [48] and Moghaddam et al. [49] stated increased TC and LDL in rodents treated with BPA, along with weight gain and fat accumulation. Moghaddam et al. [49] suggested that BPA exposure might induce hyperglycemia and its complications in adult male mice by induction of oxidative stress. Likewise, Fang et al. [50] demonstrated that BPA exposure caused increased fat and enlarged fat cells without changing body weight in hyperlipidemic rabbits.

In our study, co-administration of SeNPs or SeNPs-CV has been shown to counteract the BPA’s effects on lipid profile (Table 3). Selenium’s hypolipidemic potential is supported by multiple studies. Zhang et al. [51] demonstrated reduced lipid levels in rats that received a high-fat diet with selenium, likely by suppressing lipogenic enzymes and transporting fatty acids into mitochondria for β-oxidation. An earlier study reported that selenium was also shown to upregulate fatty acid oxidation pathways while downregulating lipogenesis, which improves lipid utilization [52]. Biogenic SeNPs lowered total lipid, triglyceride, cholesterol and LDL levels, while raising HDL levels in diabetic rats [53].

On a molecular level, BPA also influences lipid metabolism by promoting adipogenesis and lipogenesis. It upregulates key pro-adipogenic transcription factors such as sterol regulatory element-binding protein-1c (SREBP1c), which is a major regulator that initiates the de novo synthesis of fatty acids in the liver, and enhances the activity of lipogenic enzymes, including fatty acid synthase (FAS), stearoyl-CoA desaturase-1 (SCD1), and lipoprotein lipase (LPL), leading to increased lipid accumulation and adipogenesis [54]. SREBP1 is stimulated by insulin in response to carbohydrate-sufficient conditions and upregulates the gene expression of fatty acid synthesis-related enzymes such as ACC1 and FAS [55]. The carboxylation of acetyl-CoA is catalyzed by ACC1, producing malonyl-CoA, which is converted by FAS into palmitic acid, which is esterified to TG [56]. In parallel, our results showed that the transcriptional expressions of SREBP1, acetyl-CoA carboxylase (ACC1), and FAS genes in the BPA-treated group were meaningfully up-regulated as compared to the control group, indicating fatty acid synthesis. Fang et al. [50] found that BPA exposure increased adiposity and induced hypertrophy in adipocytes in both subcutaneous and visceral adipose tissue. These findings provide further insight into the possible mechanisms by which BPA influences the expression of genes involved in main pathways correlated to lipid metabolism.

Conversely, in rats co-treated with SeNPs or SeNPs-CV in the current study, the expressions of SREBP1, ACC1, and FAS genes were meaningfully downregulated in comparison with BPA group. Similarly, a preceding study found that selenium can modulate lipid accumulation in the liver of high-fat diet-fed grass carp [57]. A previous study showed that nano-selenium synthesized by Lactobacillus acidophilus similarly reduced lipid accumulation [58]. Additionally, *C. vulgaris* has been documented as having hepatoprotective, hypocholesterolaemic, anti-inflammatory, and immunomodulatory activities [59]. Bito et al. [60] reported that supplementation of *Chlorella* in diets of humans exhibited numerous valuable activities such as antioxidant, anti-hypertensive, anti-diabetic and anti-hyperlipidemic properties. These protective effects of *Chlorella* on blood lipemia have been attributed to the synergism between multiple nutrients and antioxidant compounds.

Latest studies have stated that microRNAs (miRNAs) are vital regulators of lipid metabolism. miRNAs are a class of endogenous single-stranded short RNAs that negatively control gene expression at the post-transcriptional level via binding to the 3′untranslated regions of target mRNAs [61]. Newly accumulating data have indicated that a diversity of miRNAs, principally miRNA-122, miR-370, and miR-33a/b, play vital roles in lipid metabolism [62]. Numerous crucial genes involved in lipogenesis and oxidation were found to be regulated by miRNA-122. Moreover, silencing of miRNA-122 decreased plasma levels of both TC and TG [63]. Gao et al. [64] reported that plasma levels of lipometabolism-related miRNA-122 were expressively increased in hyperlipidemia patients, and the levels of miRNA-122 were positively associated with total cholesterol, triglycerides, and LDL-C levels in hyperlipidemia. These findings are in line with our results, where the expression level of the miRNA-122 gene was significantly increased in BPA group and significantly downregulated in both SeNPs and SeNPs-CV groups. While the biogenic selenium nanoparticles have a better effect as compared to the SeNPs group, suggesting a synergistic effect of Se and *C. Vulgaris*. A previous study reported that the addition of selenium to the diet meaningfully affects the expression of miRNAs, and miRNAs play an important role in the antioxidant activities of selenium and selenium-related diseases [65]. Additionally, Nano-selenium synthesized by Lactobacillus acidophilus also reduced lipid accumulation [58].

A prior study described that BPA is a strong endocrine disruptor that alters adipose tissue function by disturbing adipokines such as leptin and adiponectin [42]. Leptin, which rises with increased fat mass, is typically elevated in obesity [66], whereas adiponectin enhances insulin sensitivity and is usually reduced in obesity and Type 2 diabetes [67]. In this study, BPA exposure increased leptin and decreased adiponectin (Fig. 7), along with greater body weight gain (Table 2), indicating disrupted adipokine balance and metabolic impairment. Elevated leptin in obesity often reflects leptin resistance, where impaired hypothalamic signaling and reduced leptin transport across the blood–brain barrier limit its appetite-suppressing effects [68, 69].

In contrast, our study revealed that supplementation with selenium nanoparticles (SeNPs) or (SeNPs-CV) markedly alleviated changes in adipokine balance induced by BPA. Decreased leptin level is typically linked to decreased body fat and contributes to energy homeostasis. These results come in line with data from Zhang et al. [51], who stated that selenium supplementation reduced lipid levels through suppression of lipogenic enzymes. *Chlorella vulgaris (CV)*, a unicellular green alga rich in polyunsaturated fatty acids, proteins, phospholipids, soluble fibers, and antioxidants (vitamins C, E, β-carotene), also exhibits promising hypolipidemic effects [70].

As reported in a former study, the liver is the principal and most vital organ where the metabolism of BPA occurs. As a result, the liver may be more susceptible to small BPA doses than other organs. BPA is commonly metabolized by the CYP2C cytochrome in the liver [71], which is the chief cause of interference in the redox cycle along with free radicals formation [72]. Hassan et al. [4] demonstrated that BPA generates ROS and reduces the antioxidant gene expression, affecting oxidant/antioxidant balance that causes hepatotoxicity and induces liver damage at the dose of 10 mg/kg. Oxidative stress, caused by an imbalance in the body’s redox homeostasis, leads to significant damage to cellular structures, including nucleic acids, proteins, carbohydrates, and lipids. Our findings revealed that BPA increased MDA, a marker of lipid peroxidation and oxidative stress, while antioxidant indices, including GSH, GPx and SOD, significantly decreased, indicating oxidative damage in the liver (Fig. 8), in line with Akash et al. [73]. This effect may also result from enzyme inactivation triggered by excessive ROS production in mitochondria and microsomes [74]. Additionally, SOD, a group of metalloenzymes, is responsible for dismutation of superoxide radicals and supporting immune function [75]. An earlier study stated that selenium efficiently enhances antioxidant activity against atrazine-induced oxidative stress in rats by raising hepatic glutathione, GPx, and SOD activity [76]. Furthermore, other studies have confirmed selenium’s crucial protective role in oxidative stress-related liver damage [77, 78]. In the present study, co-treatment with selenium nanoparticles (SeNPs) and SeNPs-CV showed a significant improvement in SOD, GPx, GSH, and MDA levels. These findings align with those stated by El-Borady et al. [79] and Pérez Gutiérrez et al. [80], supporting their role in reducing BPA-induced oxidative stress in liver tissues. Our results demonstrated that SeNPs alleviated oxidative stress, with SeNPs-CV being more effective in increasing SOD activity compared to the SeNPs group. These outcomes emphasize SeNPs-CV’s ability to counteract oxidative stress and lipid damage caused by BPA. This improvement may be ascribed to the synergistic antioxidant activities of selenium and *Chlorella vulgaris*. A previous study reported that flavonoid-coated SeNPs also enhanced antioxidant defenses (SOD, CAT, GPx), reduced lipid peroxidation, and protected β-cell architecture even under hyperglycemic stress [81].

In summary, BPA affects lipid metabolic pathways via alterations in the transcriptional levels of many genes involved, while inhibiting hepatic β-oxidation of fatty acids. This impairs the timely removal of excess lipids, resulting in their accumulation in the liver, which leads to subsequent intense oxidative stress in the liver, causing damage to liver cells.

In addition to hormonal disruption, BPA negatively influences inflammatory signaling pathways, including the NF-κB pathway, contributing to metabolic dysfunction and oxidative stress. Higher BPA doses, associated with elevated nitric oxide and increased phosphorylation of NF-κB and p38 mitogen-activated protein kinases (p38 MAPK), upregulate pro-inflammatory cytokines and nitric oxide synthase 2 (NOS2), indicating a strong link between redox imbalance and inflammation in BPA-exposed testes [82]. Similar findings were reported in *Gobiocypris rarus*, where BPA increased TNF-α and interleukin-1 beta (IL-1β) protein levels and elevated p38 MAPK and c-Jun N-terminal kinase (JNK) transcripts [83]. Since adipokines also function as inflammatory mediators, our study evaluated inflammatory markers following BPA exposure, including serum leptin, adiponectin, TNF-α, and total NF-κB p65 levels. BPA exposure led to a marked increase in leptin, TNF-α, and total NF-κB levels, reflecting an inflammatory response. On the contrary, adiponectin, known as anti-inflammatory, was meaningfully diminished in the BPA group, supporting the notion that BPA promotes inflammation, consistent with Akash et al. [73]. Variations in NF-κB p65 levels are discussed as indicative observations and not as conclusive evidence of NF-κB pathway activation. To confirm the role of NF-κB signaling, further research is needed, including phosphorylated NF-κB and/or its nuclear localization assessments.

However, SeNPs and SeNPs-CV treatment in the current study significantly mitigated the BPA-induced inflammatory response. Our current results suggest that Se and CV can restore the balance of adipocytokines, alleviate the inflammatory effects of BPA and demonstrate its anti-inflammatory potential against BPA-induced toxicity. These findings agree with a former study that reported that selenium nanoparticles have garnered interest as potent anti-inflammatory agents [84]. Their mechanism involves modulation of cytokine production, TNF-α, and interferon gamma (IFN-γ) through the suppression of inflammatory mediators such as toll-like receptors (TLRs) and the NF-κB [85]. According to a previous study in animal models, SeNPs also alleviated colonic inflammation by suppressing NF-κB nuclear translocation and reducing pro-inflammatory cytokines [86]. Additionally, *Chlorella vulgaris* was shown to suppress the NF-κB/IL-1β inflammatory pathway, lowering pro-inflammatory cytokines and neutrophil infiltration in hepatic ischemia-reperfusion injury [87]. Green-synthesized SeNPs from plant extracts displayed robust anti-inflammatory properties, attributed to the combined antioxidant effects of selenium and bioactive natural compounds like flavonoids and phenols [88].

Liver biomarkers such as ALT, AST and albumin serve as key indicators of liver function and hepatocellular integrity. Elevated levels of ALT and AST typically reflect hepatocyte membrane leakage and hepatic injury [89], while alterations in albumin levels are indicative of impaired synthetic capacity of the liver [90]. Previous reports have associated BPA toxicity with oxidative stress-mediated hepatocellular injury [91, 92] and testicular dysfunction [93]. In the current study, exposure to BPA caused a noteworthy increase in serum AST and ALT activities and a noteworthy reduction in serum albumin levels in relation to the control group. Even though there were statistically significant alterations in ALT and AST, their absolute levels were within the typical biological reference ranges. Because the study is based on comparisons with the control group, the results are indicative of a treatment-related effect, most likely showing mild hepatic changes rather than evident liver damage. However, treatment with selenium nanoparticles (SeNPs) or (SeNPs-CV) for 45 days markedly reduced ALT and AST activities and increased albumin levels in the BPA group, suggesting a protective role against liver injury. These results support the notion that SeNPs confer hepatoprotection by restoring membrane stability and reducing enzyme leakage into the bloodstream and restoring liver marker levels to near normal [80]. These improvements in hepatic health are possibly ascribed to the antioxidant and anti-inflammatory activities of selenium [53] and *Chlorella vulgaris* [94]. SeNPs encapsulated with flavonoids improved serum lipid profiles, in part through enhancing antioxidant defence systems and supporting liver function [95].

**Fig. 11:**
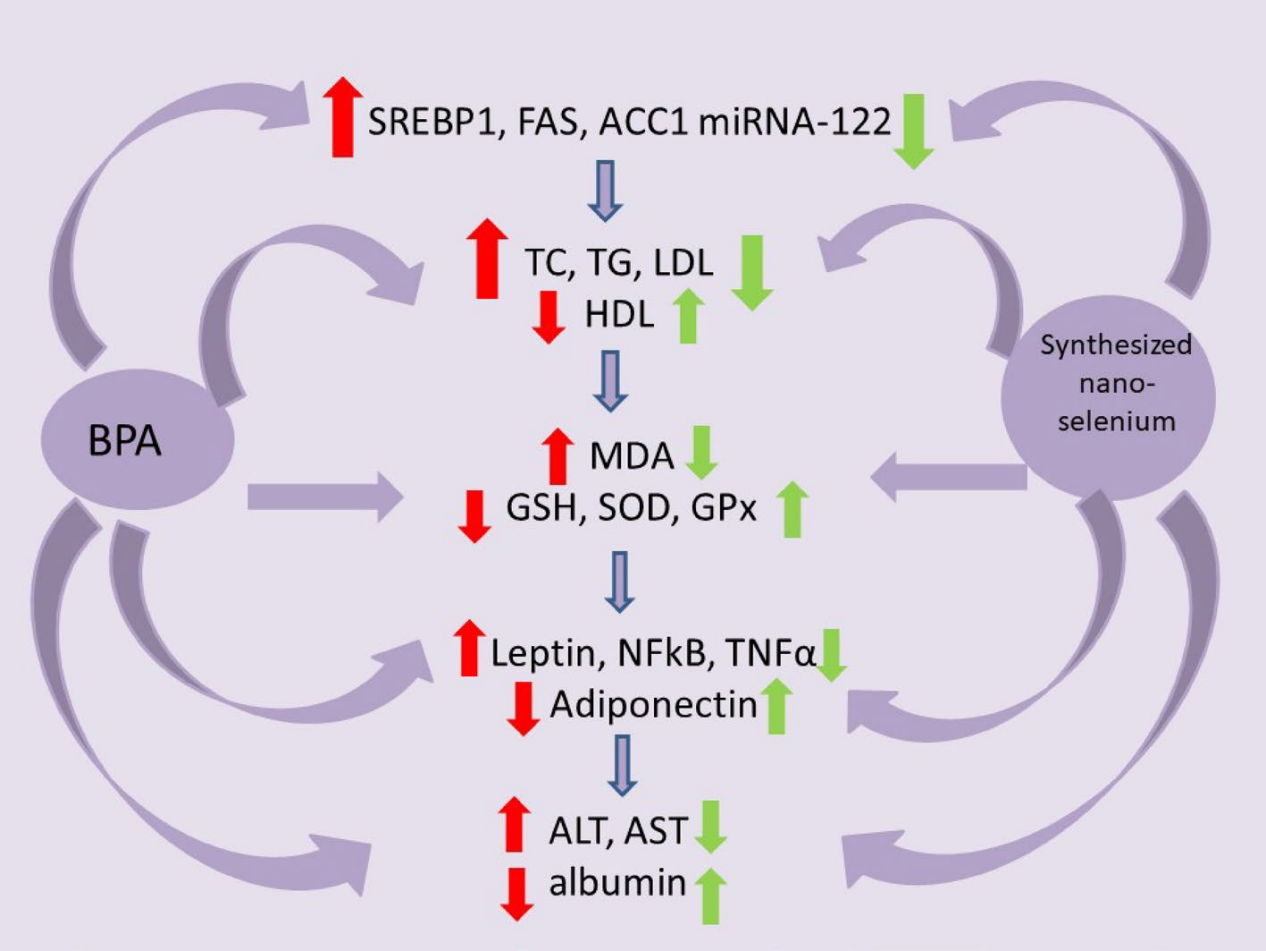
Infographic summary of the proposed hepatoprotective mechanisms: SeNPs and SeNPs-CV exert hypolipidemic and antioxidant effects that reduce oxidative stress and inflammatory signaling, thereby ameliorating hepatic dysfunction. Fatty acid synthesis-related genes: Sterol regulatory element binding protein-1 (SREBP1), fatty acid synthase (FAS), acetyl-CoA carboxylase (ACC1), microRNA-122 (miRNA-122). Lipid profile: Total cholesterol (TC), Triacylglycerols (TG), High-density lipoprotein (HDL), Low-density lipoprotein (LDL). Oxidative stress: Reduced Glutathione (GSH), Malondialdehyde (MDA), Superoxide Dismutase (SOD), Glutathione peroxidase (GPx). Inflammatory mediators: Leptin, Adiponectin, nuclear factor kappa-B (NF-κB), Tumor Necrosis Factor-alpha (TNF-α). Liver biomarkers: Albumin, Alanine Aminotransferase (ALT), Aspartate transaminase (AST). Green arrows: positive increase or decrease. Red arrows: negative decrease or increase

Our results revealed that these biochemical alterations are consistent with the histopathological findings observed in BPA-treated animals. The control group exhibited normal liver architecture. In contrast, BPA exposure caused clear hepatic damage, including hydropic degeneration of hepatocytes, widened sinusoids, and dilation of the central vein and portal areas, consistent with impaired liver function. BPA has been identified to have adverse effects on liver function [96], which was mainly attributed to increasing ROS [97]. The histopathological improvements observed in the SeNPs and SeNPs-CV groups align with biochemical data and previous studies, demonstrating reduced oxidative stress and restored hepatic organization (Fig. 10). The protective role of selenium is supported by its ability to counteract ROS [98]. Additionally, *Chlorella vulgaris* has been shown to protect against drug-induced liver injury, confirming its potent antioxidant effects [94]. This better regenerative effect of SeNPs-CV is likely due to the combined antioxidant properties of Se nanoparticles and the phenolic compounds in *Chlorella vulgaris*, consistent with previous findings of Pérez Gutiérrez et al. [80] (Fig. 11).

The protective effects of SeNPs and SeNPs-CV should not be directly generalized to real-world conditions; instead, confirmation at environmentally relevant doses is necessary because the BPA dose used in this study represents a high-dose toxicological model that does not reflect typical human exposure. Although the results point to oxidative stress and lipogenic stress pathway regulation, these mechanisms are inferred rather than proven. However, additional studies involving pathway inhibition, protein expression analysis, and functional assays are necessary to confirm causal mechanisms, in addition to evaluating physicochemical characterization, long-term safety and optimizing dosing strategies for clinical application.

## Conclusion

The current study highlights that CV serves as both a reducing and capping agent in the biosynthesis of SeNPs-CV, producing a formulation with enhanced properties. Both SeNPs and SeNPs-CV modulated lipid profile, reduced oxidative stress and inflammation, balanced leptin and adiponectin levels, restored hepatic gene expressions, and alleviated liver histopathological changes. Green synthesis offers an eco-friendly method for SeNP production, eliminating the need for chemical agents. Algal extracts, rich in bioactive compounds, serve as reducing and stabilizing agents during the synthesis of nanoparticles, contributing to the antioxidant, anti-inflammatory, and insulin-sensitizing properties of SeNPs, further enhancing the biomedical applications of SeNPs. The study is among the first to explore the metabolic benefits of biogenic SeNPs-CV in BPA-induced metabolic disorders, suggesting they may be promising green therapeutic agents. Although SeNPs-CV appear to mitigate BPA-induced metabolic and oxidative disturbances, the proposed mechanisms are preliminary. Further research is needed to validate molecular pathways, assess long-term safety and efficacy, and confirm causal relationships through advanced mechanistic and physicochemical analyses.

## Data Availability

The data that support the findings of this study are contained within the article.
